# Pneumococcal conjugate vaccine effectiveness against hypoxemia in children with suspected pneumonia in Kenya; analysis from a real-world sentinel surveillance platform

**DOI:** 10.1371/journal.pone.0351500

**Published:** 2026-06-26

**Authors:** Fredrick Mutisya, George O. Agogo, Jared Opudo, Barbara Jepkorir, Athman Mwatondo, Daniel Langat, Peninah Munyua, Duncan Chege, Jonas Z. Hines

**Affiliations:** 1 Kenya Field Epidemiology and Laboratory Training Program, Nairobi, Kenya; 2 U.S. Centers for Disease Control and Prevention, Nairobi, Kenya; 3 ICAP at Columbia University, Nairobi, Kenya; 4 Africa Field Epidemiology Network (AFENET), Nairobi, Kenya; 5 Kenya Ministry of Health, Nairobi, Kenya; IAVI, UNITED STATES OF AMERICA

## Abstract

The 10-valent pneumococcal conjugate vaccine (PCV-10) has reduced the burden of pneumococcal disease in children. Integrated facility-based sentinel surveillance (IFBS) monitors a range of common causes of febrile illnesses in Kenya. We used this system to assess the real-world vaccine effectiveness (VE) of PCV-10 against hypoxemia among children with suspected pneumonia in Kenya. We analysed IFBS data collected from 13 sites across Kenya from 2017 to 2024. We included children aged 6 weeks to 59 months with reported fever or temperature ≥38.0°C, respiratory features consistent with suspected pneumonia, and a copy of their vaccination records (i.e., child health booklet). We excluded children with a positive test result indicating a non-Pneumococcal aetiology (i.e., positive result on a multiplex PCR, malaria microscopy or rapid diagnostic test, or SARS-CoV-2 PCR). We assessed the association of vaccination status with hypoxemia, defined as an oxygen saturation of ≤90%, adjusting for age, sex, and year. VE was calculated as 1 minus the odds ratio of hypoxemia among children with suspected pneumonia calculated using generalized estimating equations to account for clustering by site. Overall, 3,533 children were analysed, with 94% being up to date with PCV-10 vaccination. The median patient age was 11 months (interquartile range: 6–17). Hypoxemia was observed in 37% of fully vaccinated children, 41% in partially vaccinated children, and 52% in unvaccinated children (χ² test, p = 0.03). The adjusted VE for full vaccination against hypoxemia in children with suspected pneumonia was 39% (95% confidence interval: 6–61%). Among children with suspected pneumonia in Kenya, PCV-10 vaccination was associated with reduced hypoxemia. The PCV-10 coverage in this dataset was high, potentially reflecting a robust routine vaccination effort since PCV-10 was introduced in Kenya in 2011. High-quality surveillance data can be used to provide real-world evidence to support routine immunization programs.

## Introduction

Respiratory disease is a major cause of morbidity and mortality in Kenya [1] with pneumonia contributing to 8,000–10,000 deaths annually among children aged under 5 years in Kenya in 2023 [2]. *Streptococcus pneumoniae* is a common cause of bacterial respiratory infections [3]. In a multicountry study that included Kenya done from 2011 to 2014, *S. pneumoniae* was a common cause of bacterial pneumonia, with an aetiologic fraction of 5% after accounting for nasopharyngeal carriage [4]. Additional data from Kenya support *S. pneumoniae* as a common bacterial cause of pneumonia [5,6].

To address the burden of respiratory infections in Kenya, the government introduced the pneumococcal conjugate vaccine with antigens to ten *S. pneumoniae* serotypes (PCV-10) into its routine immunization program in January 2011, with a three infant doses and no booster (“3 + 0”) schedule [7]. PCV-10 is currently available in two formulations: Synflorixfrom GSK and Pneumosil from Serum Institute of India [8,9], with Kenya using the latter brand since 2022.

A study done in an urban informal settlement in Nairobi and a rural village in Siaya County demonstrated a reduction in pneumococci colonization among children aged <5 years following introduction of PCV-10 [10]. These results were similar to those in Kilifi County, where the prevalence of vaccine-type pneumococcal serotypes declined after introduction of PCV-10 [11]. In Kilifi, the annual incidence of admissions for clinically defined pneumonia declined by 27% after PCV-10 introduction [12]. Additionally, introduction of PCV-10 among children in western Kenya was associated with a decline in pneumococcal pneumonia incidence in adults [13]. Despite evidence from Kenya demonstrating reductions in vaccine-type pneumococcal carriage and pneumonia following PCV-10 introduction, these previous evaluations did not include pulse oximetry measurement to classify pneumonia severity and were conducted between 2011–2014 so may not reflect more recent evidence of public health impact or evolving serotype dynamics. While VE studies might be best done soon after vaccine introduction when coverage is below 80% [14], providing data from real world conditions on vaccine performance can help provide evidence to sustain vaccination programs. Kenyan studies have been done in small geographic areas with in-depth surveillance platforms [9–13]. We sought to address these gaps by leveraging an infectious disease sentinel surveillance platform during 2017–2024, applying a hypoxaemia‐based case definition to assess PCV‐10 effectiveness among children with suspected pneumonia across diverse Kenyan regions.

## Materials and methods

### Study site

**Fig 1:**
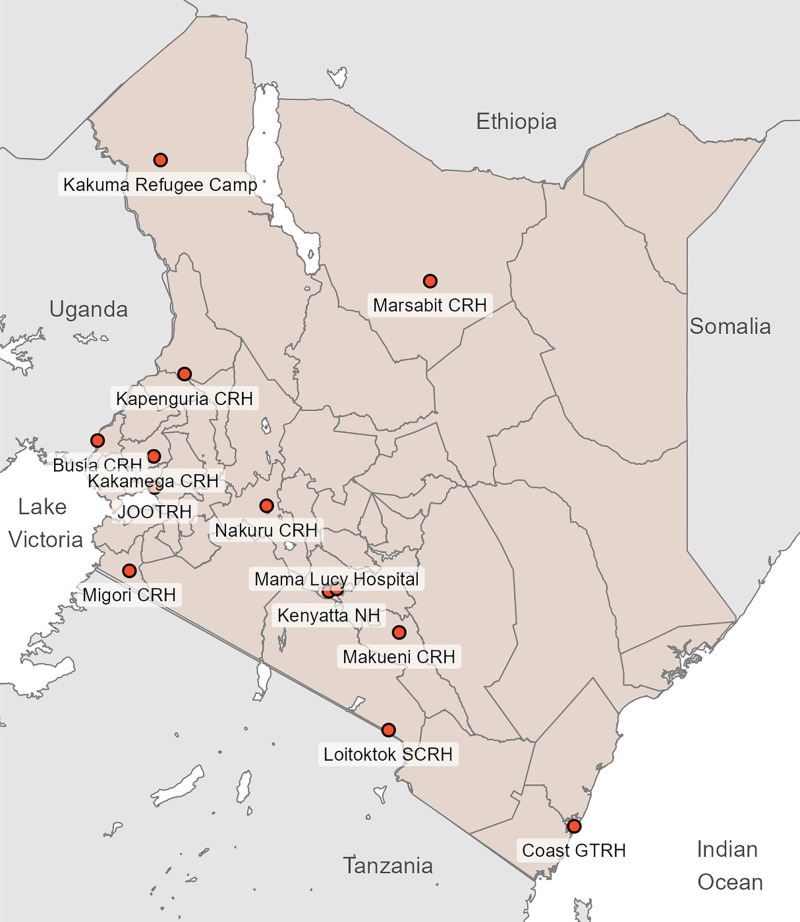
Integrated facility-based surveillance sites included in pneumococcal conjugate vaccine effectiveness analysis, Kenya, 2017-2024. This map was created with shapefiles downloaded from the following sources: 1) World: https://www.naturalearthdata.com/?s=kenya; 2) Kenya: https://data.humdata.org/dataset/cod-ab-ken; and 3) Lakes: https://energydata.info/dataset/africa-water-bodies.

We used data from the Integrated Facility-based Surveillance (IFBS) system, which is an infectious disease sentinel disease surveillance platform in Kenya [15]. The platform began in 2017 as an acute febrile illness (AFI) surveillance system in four health facilities and has expanded to cover additional syndromes (severe acute respiratory infection [SARI], mortality) and now includes 12 sites; some sites were discontinued and this analysis included data from 13 sites total (Fig 1).

### Sentinel surveillance protocol

Each weekday morning, trained surveillance officers reviewed hospital admission logs from the previous 24 hours for pediatric and adult medical wards at participating facilities. All new admissions were screened for eligibility criteria using information in the medical record. Patients were eligible for enrolment if they had AFI, defined as a measured temperature ≥38.0°C for ≤14 days, or SARI, defined as a reported fever or measured temperature ≥38.0°C and cough for ≤10 days that required hospitalization. Because surveillance was not conducted on weekends, surveillance officers also reviewed weekend admissions on Monday mornings and approached eligible patients for enrolment if they had received antimicrobial therapy for less than 24 hours. Patients who died before they could be approached by surveillance officers were not eligible for enrolment. Eligible patients were approached for informed consent (and for minors, guardian consent and assent as applicable) and enrolment into the surveillance system study. Additionally, at some sites, recruitment for the AFI module was done at outpatient department clinics for persons aged ≥13 years.

### Data collection

Once enrolled, the surveillance officer interviewed the patient and collected data from medical records using a standardized digital form and uploaded to an encrypted server each day. Data collected included demographics, household socioeconomic indicators, medical history including routine vaccination either by verbal report or transcribed from the Ministry of Health child health booklet, clinical presentation, vital signs, anthropometry, physical exam, laboratory results, and clinical management (e.g., supplemental oxygen, antibiotics). Additionally, the patients’ clinical outcome (e.g., discharged, transferred, died) was collected at the end of their hospitalization. Pulse oximeters (Masimo Rad-5 and Lifebox®) captured the haemoglobin oxygen saturation levels and were calibrated quarterly. Patients with AFI who had an undifferentiated cause of their fever had whole blood sample collected for testing with a multiplex polymerase chain reaction (PCR). Undifferentiated fever was defined as AFI without evidence of lower respiratory tract infection (cough or shortness of breath plus tachypnea or abnormalities on respiratory examination), diarrhea (≥3 loose stools in a 24-hour period), or another focus of fever based on history and physical examination (e.g., meningitis, skin/soft tissue infection). Patients with SARI had a nasopharyngeal swab collected for SARS-CoV-2 PCR testing.

### Laboratory testing

Blood samples were drawn under aseptic conditions. Malaria diagnostics included both smear microscopy and SD Bioline® HRP-2 combo rapid diagnostic test. For patients who met undifferentiated fever criteria, whole blood samples were stored at −20˚ C for up to seven days and transported to the US Centers for Disease Control and Prevention (CDC)-supported laboratory at the Kenya Medical Research Institute (KEMRI) in Nairobi for PCR testing using TaqMan Array Card (TAC) multiplex PCR test [ThermoFisher]. From May 2017 to November 2020, the TAC assay included targets for *Bartonella*, *Brucella*, *Coxiella burnetii*, Crimean-Congo haemorrhagic fever virus, chikungunya virus, dengue virus, Ebola virus, Ebola (Bundibugyo) virus, Ebola (Sudan) virus, hepatitis E virus, Lassa virus, *Leishmania* spp., *Leptospira* spp., Marburg virus, Nipah virus, O'nyong'nyong virus, Plasmodium, *Rickettsia* spp., Rift Valley Fever virus, *Salmonella* spp., *Salmonella* Typhi, HIV I, HIV II, *Trypanosoma brucei*, West Nile virus, *Yersinia pestis*, yellow fever virus, and Zika virus. The TAC assay was updated in November 2020 to add additional targets for: *Burkholderia pseudomallei*, *Orientia tsutsugamushi*, Oropouche virus, *Plasmodium falciparum*, *Plasmodium vivax*, *Streptococcus pneumoniae*, and *Salmonella* Paratyphi A. TAC PCR was intended for surveillance and was not approved for the diagnoses of patients for clinical purposes. From June 2021 onward, nasopharyngeal swabs were tested for SARS-CoV-2 by PCR per national guidelines at KEMRI laboratory in Nairobi.

### Data analysis

Data were analysed from the 13 sentinel IFBS health facilities across Kenya, which were not constant during the analysis period (S1 Table). We analysed data from enrolled children age ≥ 6 weeks to ≤59 months with at least one respiratory sign or symptom including cough, difficulty breathing, chest in-drawing, grunting, stridor, nasal flaring or crackles, which we defined as suspected pneumonia. Persons aged ≥60 months were not asked about vaccination status and children aged <6 weeks were not yet eligible for PCV-10. We excluded patients with missing vaccination information or vaccination history that was by verbal report alone. We also excluded patients with a positive result on TAC (except S. *pneumoniae*), malaria microscopy or rapid diagnostic test, or SARS-CoV-2 PCR since they had a probable non-S. *pneumoniae* diagnosis.

PCV-10 vaccination status was classified as per Kenya’s Expanded Program on Immunization [16]. A patient was unvaccinated if they had received no PCV-10 doses, partially vaccinated if they had received ≥1 dose but did not meeting age-appropriate schedule of 6 weeks, 10 weeks and 14 weeks (e.g., 1 PCV-10 dose received by 9 months-old), and fully vaccinated if they had received all recommended doses for their age. Malnutrition was defined by a patient’s mid-upper arm circumference (MUAC) of <12.5 cm. Hypoxemia was defined as a child with an SpO_2_ ≤ 90% on ambient air or who was using supplemental oxygen at admission.

De-identified data were downloaded from a REDCap database into Microsoft Excel on September 6, 2024, and analysed in R [R Core Team, 2024, version 4.3.2] using tidyverse, geepack, and lme4 packages. Chi-square test was done to compare categorical variables. Effect modification was tested using the Breslow-Day test. We developed a directed acyclic graph (S1 Fig) to conceptualize confounder variables rather than rely on a prespecified p-value threshold [17]. Multiple logistic regression with generalised estimating equations (GEE) was done to adjust for age, sex, and year accounting for potential correlation within clusters (surveillance site). GEE used an exchangeable correlation structure to obtain more robust standard errors [18]. Vaccine effectiveness (VE) was calculated as 1 – odds ratio (OR), and 95% confidence intervals (CIs) were generated via the delta method.

Sensitivity analysis was done using routine logistic regression and a general linear mixed model (GLMM). GLMM with random intercepts for site accounted for between-facility heterogeneity [19]. We also did a sensitivity analysis to assess the impact of our inclusion criteria, calculating the crude VE along each step as we filtered the data. To assess for the risk of bias we assessed the association between two unrelated factors, PCV-10 vaccination status and malaria test results (both microscopy and RDT) and found no association to indicate systematic bias in the data.

## Ethics

During eligibility screening for enrolment in the IFBS system, the surveillance officer obtained verbal and written informed consent in English, Swahili, or a preferred local language from a parent or guardian of patients, including for data collection and biological specimens. The data were collected under the KEMRI institutional review board protocol number SERU 2980 and 4232. This activity was reviewed by CDC, deemed not research, and was conducted consistent with applicable U.S. federal law and CDC policy (See, e.g., 45 C.F.R. part 46.102(l)(2), 21 C.F.R. part 56; 42 U.S.C. §241(d); 5 U.S.C. §552a; 44 U.S.C. §3501 et seq.).

## Results

From May 27, 2017 to September 5, 2024, out of 151,546 patients screened, 23,774 (16%) were eligible, of whom 17,625 (74%) were enrolled (percent enrolled range: 58% [2017] to 82% [2021]). Out of 17,625 people enrolled, 12,614 (72%) were age 6 weeks through 59 months, of whom 8,343 (66%) had at least one respiratory symptom (S2 Fig). Among these, 7,202 (86%) had a negative malaria test, a negative SARS-CoV-2 test, or a negative TAC test (except *S. pneumoniae*) or no TAC test was performed. Finally, of these, we analysed data from 3,533 (49%) children who had a verifiable vaccination record (i.e., child health booklet).

The median patient age was 11 months (IQR 6–17 months), with 56% being male (Table 1). The patients’ residence spanned 35 counties, with the top three counties being Turkana (21%), Nairobi (14%), and Mombasa (13%). Household heads were mostly informally employed (i.e., casual labourers [37%] or self-employed [26%]). Twenty-nine percent of patients had received antibiotics within seven days of enrolment. Blood specimens were positive for *S. pneumoniae* on TAC PCR test in only five patients (<1%). PCV-10 coverage was high, with 94% being fully vaccinated, 4% partially vaccinated, and 2% unvaccinated.

**Table 1:** Characteristics and bivariate analysis of factors associated with hypoxemia among children aged 6 weeks to 59 months with suspected pneumonia, Kenya, 2017-2024.

Characteristic	Overall	No Hypoxemia	With Hypoxemia	OR
	(N = 3,533)	(N = 2,213)	(N = 1,320)	(95% CI)
**Age Group (months)**				
<6 months	748 (21%)	374 (17%)	374 (28%)	ref
6–11 months	1,470 (42%)	930 (42%)	540 (41%)	0.58 (0.49–0.69)
12–23 months	909 (26%)	621 (28%)	288 (22%)	0.46 (0.38–0.57)
24–59 months	406 (11%)	288 (13%)	118 (8.9%)	0.41 (0.32–0.53)
**Sex**				
Male	1,995 (56%)	1,279 (58%)	716 (54%)	ref
Female	1,538 (44%)	934 (42%)	604 (46%)	1.16 (1.01–1.33)
**Household Head Occupation (n missing: 1)**				
Casual Labourer	1,308 (37%)	718 (32%)	590 (45%)	ref
Self employed	934 (26%)	551 (25%)	383 (29%)	0.85 (0.71-1.00)
Professional	536 (15%)	334 (15%)	202 (15%)	0.74 (0.60-0.90)
Currently unemployed	754 (21%)	609 (28%)	145 (11%)	0.29 (0.23-0.36)
**Mother Education Level (n missing: 17)**				
No formal education	464 (13%)	391 (18%)	73 (5.5%)	ref
Incomplete primary school	562 (16%)	389 (18%)	173 (13%)	2.38 (1.76–3.25)
Complete primary school	643 (18%)	357 (16%)	286 (22%)	4.29 (3.21–5.79)
Incomplete secondary school	456 (13%)	294 (13%)	162 (12%)	2.95 (2.16–4.06)
Complete secondary school	857 (24%)	466 (21%)	391 (30%)	4.49 (3.40–6.00)
College/university	534 (15%)	307 (14%)	227 (17%)	3.96 (2.94–5.39)
**HIV status of the child (by report) (n missing: 1,064)**				
Positive	16 (1%)	11 (1%)	5 (1%)	0.74 (0.23–2.05)
Negative	2,453 (99%)	1,523 (99%)	930 (99%)	ref
Nutritional Status* (n missing: 973)				
Not malnourished	1,897 (74%)	1,247 (76%)	650 (70%)	ref
Malnourished	663 (26%)	390 (24%)	273 (30%)	1.34 (1.12-1.61)
**Recent Antibiotic Use (≤7 days) (n missing: 226)**				
No	2,299 (70%)	1,561 (74%)	738 (61%)	ref
Yes	1,008 (30%)	537 (26%)	471 (39%)	1.86 (1.59–2.16)
**PCV-10 Vaccination Status**				
Unvaccinated	65 (1.8%)	31 (1.4%)	34 (2.6%)	ref
Partial	138 (3.9%)	82 (3.7%)	56 (4.2%)	0.62 (0.34–1.13)
Full	3,330 (94%)	2,100 (95%)	1,230 (93%)	0.53 (0.33–0.87)
**Outcome (n missing: 269)**				
Discharged home (Stable)	3,082 (95%)	2,005 (97%)	1,077 (90%)	ref
Transferred to other hospital	14 (<1%)	6 (<1%)	8 (<1%)	2.49 (0.86–7.57)
Absconded	23 (<1%)	20 (1%)	3 (<1%)	2.48 (0.07–0.82)
Died	145 (4%)	35 (1%)	110 (9%)	5.86 (4.02–8.75)

CI: confidence interval; MUAC: mid-upper arm circumference: PCV: pneumococcal conjugate vaccine.

* Malnutrition defined by the MUAC cut-off value of 12.4 cm if aged >6 months and 11.0 cm if aged 6 weeks to 6 months.

Hypoxemia was greater among unvaccinated children with suspected pneumonia compared with fully vaccinated children (52% vs 37%; χ ^2^, p = 0.03). Compared with unvaccinated children, crude vaccine effectiveness (VE) against hypoxemia was 47% (95% CI: 13–67%) for fully vaccinated children and 38% (95% CI: –13–66%) for partially vaccinated children (Table 2).

**Table 2:** PCV-10 vaccine effectiveness estimates against hypoxemia in children aged 6 weeks to 59 months with suspected pneumonia in the integrated facility-based sentinel surveillance system in Kenya, 2017-2024.

	Crude VE, % (95% CI)	Adjusted VE,* % (95% CI)		
		Logistic regression	GEE	GLMM
PCV-10 vaccination status				
Full	47 (13, 67)	42 (2, 65)	39 (6, 61)	44 (3, 68)
Partial	38 (−13, 66)	26 (−38, 60)	−1 (−71, 40)	−22 (−140, 38)
Unvaccinated	–	–	–	–

* Adjusted for age, sex, and year. Additionally, the GEE and GLMM models adjusted for clustering by surveillance site.

CI: confidence interval; GEE: generalized estimating equation; GLMM: generalized linear mixed model; PCV: pneumococcal conjugate vaccine; VE: vaccine effectiveness.

In the sub-group analysis stratified by recent antibiotic use, VE was 43% (95% CI −14–71%) in children without recent antibiotics and 53% (95% CI −27–84%) among those who had received antibiotics in the preceding week (Table 3); the Breslow–Day test for homogeneity for recent antibiotic use was not statistically significant (χ² = 0.13, df = 1, p = 0.72). When stratified by MUAC, the VE was statistically significant in children with normal MUAC (i.e., not malnourished) (74% [95% CI: 3–94%) but not in malnourished children (4% [95% CI: −179–65%]); the Breslow–Day test for homogeneity for nutritional status was not statistically significant (χ² = 3 df = 1, p = 0.08).

**Table 3:** PCV-10 vaccine effectiveness estimates for hypoxemia in children aged 6 weeks to 59 months with suspected pneumonia, stratified by potential effect modifiers, Kenya, 2017-2024.

Effect Modifier	Stratum	VE, % (95% CI)	Breslow-Day Test p-value
Antibiotic use in past 7 days	Yes	53 (−27, 84)	0.718
	No	43 (−14, 71)	
Mid-upper arm circumference	≥12.5 cm	74 (3, 94)	0.083
	<12.5 cm (malnourished)	4 (−179, 65)	

CI: confidence interval; PCV: pneumococcal conjugate vaccine; VE: vaccine effectiveness.

After adjusting for child age, sex, and year of enrolment and accounting for potential within-site correlation with an independence working matrix (GEE), full vaccination remained protective (VE = 39%; 95% CI: 6–61%) (Table 2). In the sensitivity analysis using GLM, up-to-date PCV-10 vaccination had an adjusted VE of 42% (95% CI: 2–65%) against hypoxemia compared with unvaccinated children with suspected pneumonia. Introducing a random intercept for surveillance site (GLMM) had an adjusted VE for full vaccination of 44% (95% CI: 3–68%). The additional sensitivity analysis conducted based on the inclusion criteria is in S2 Table; the absolute difference in vaccine effectiveness (|ΔVE|) between each step and the final dataset ranged from 7 to 12 percentage points.

## Discussion

PCV-10 vaccination was associated with reduced hypoxemia among children with suspected pneumonia in a sentinel surveillance system in Kenya. Given the burden of respiratory disease in Kenya, our findings demonstrate the importance of this vaccine in the routine immunization program and further strengthens the existing evidence for this preventive intervention. Data on PCV-10 effectiveness specifically against hypoxemic endpoints in sub-Saharan Africa remain scarce. Our study findings are in line with an observational study from Malawi, which reported an 47% overall reduction in hypoxemic pneumonia following introduction of 13-valent pneumococcal conjugate vaccine [20].

Our findings add to the evidence of effectiveness of PCV-10 against pneumonia. A study in Asia that used the 2013 WHO definition of pneumonia found that PCV-13 has an adjusted VE of 35% against hypoxic pneumonia [21] with a similar study in Papua New Guinea having a 28.7% reduction in hypoxic pneumonia [22]. A PCV manufacturer’s phase 3 trial conducted in Argentina, Panama, and Colombia, reported an intent-to-treat vaccine efficacy of 18% and a per-protocol VE of 22% against pneumonia as defined by the World Health Organization (WHO) [23]. In Kenya, a slightly higher VE of 27% against clinically defined pneumonia was reported from an interrupted time-series analysis in Kilifi County, Kenya (95% CI 3–46%) [12]. This study relied on the WHO clinical case definition and did not incorporate oxygen saturation measurement by pulse-oximetry, a methodological distinction that plausibly attenuated its VE estimate by including milder respiratory illnesses. The present findings reinforce the already established diagnostic and epidemiological importance of pulse oximetry in pneumonia [24].

PCV-10 coverage was high, likely reflecting the positive impact of government-led childhood immunization programs. The PCV-10 coverage in the surveillance cohort was marginally higher than the 91% administrative coverage reported nationally for 2023 [25]. Such overestimation might be expected in facility-based surveillance, which preferentially captures children who are more likely to engage with preventive services and therefore to be fully vaccinated [26]. Despite the very high coverage in this dataset, the analysis highlights a real-world use of surveillance data in generating evidence to support public health interventions like vaccination programs [27].

IFBS also supports Kenya’s notifiable disease surveillance system, Integrated Disease Surveillance and Response (IDSR) [28], by helping to identify the cause of common infectious syndromes, detect outbreaks, and inform public health decision makers. Testing done as part of IFBS covers >10 notifiable pathogens under IDSR, expanding Kenya’s ability to identify many infectious disease threats. Additionally, IFBS has confirmed expansion of chikungunya virus in Kenya, which can inform vaccination policy for travelers [29], and this platform identified an outbreak of Rift Valley Fever virus infections in 2024 [30]. Furthermore, CDC-supported surveillance platforms can be leveraged to better understand infectious disease epidemiology and preventive strategies, such as for malaria [31], in Kenya. Here, we have further demonstrated how high-quality surveillance data can be used to provide real-world evidence to support a routine immunization program.

Subgroup analysis was done on recent antibiotic use as a possible effect modifier due to the convergent impact of vaccination and antibiotic use [32]. Our subgroup analysis indicate that recent antibiotic exposure did not modify the protective effect of the vaccine in this dataset. Because the surveillance system only captures children sick enough to reach the hospital, patients who took antibiotics and who recovered at home are missing. This selection bias means the “recent antibiotics” group in our data is skewed toward severe, antibiotic-refractory pneumonia. While malnutrition has long been recognized as a modifier of vaccine response, the data on vaccine effectiveness of PCV-10 in this subpopulation has been limited [33]. In well‐nourished children, we saw a protective effect of the vaccine. Studies from South Africa and Kenya have demonstrated variable immune effects of pneumococcal conjugate vaccination based on the recipient’s nutritional status [34,35].

Across the three complementary models—logistic regression, GEE, and GLMM—the direction and magnitude of the association between full PCV-10 vaccination and reduced hypoxemia in children with suspected pneumonia were consistent despite different methods for handling site clustering. Partial vaccination was not protective in any model, and its confidence intervals spanned the null, which may be attributable to the small sample size in this group.

Our analysis had several limitations. Recruiting patients the morning after admission likely excluded those who died soon after presentation to the health facility, biasing toward less severe cases in the surveillance system. Although VE findings were statistically significant for full vaccination, the confidence intervals were wide, likely because of the small proportion of patients who were unvaccinated. Only five children had *S. pneumoniae* detected in their blood after the *S. pneumoniae* target was added to the TAC PCR test in November 2020 and there was not a direct test for pneumococcus confined to lung compartment; hence, despite excluding malaria, SARS-CoV-2, and non-pneumococcal TAC-positive case, some children included in the analysis might have had pneumonia from another causes that would have biased the VE estimate toward the null. The surveillance system uses a sentinel approach and skews toward larger hospitals, which tend to be in more urban areas, which could affect the representativeness of our findings. Furthermore, site openings/closures and a surveillance pause early in the COVID-19 pandemic created temporal gaps. Another temporal limitation of this study is that the VE analysis was conducted several years after the introduction of PCV-10, during a period of high vaccine coverage. Nevertheless, we contend that the value of obtaining real-world evidence outweighs this limitation. Lastly, some covariates like HIV status were self-reported, which may have introduced measurement error (i.e., misclassification) and missing data.

In conclusion, maintaining high PCV-10 coverage and closing the remaining PCV-10 vaccination gap can maximize the public health gains demonstrated here and in other studies. Furthermore, IFBS and other surveillance platforms like the population-based infectious disease surveillance system can be leveraged to support public health decision-making in Kenya.

## Supporting information

S1 Fig Directed acyclic graph of the relationship between pneumococcal conjugate vaccine and hypoxemia, Kenya, 2017–2024. (PDF)

S2 Fig Study flow chart for vaccine effectiveness study of PCV-10 using sentinel surveillance data in Kenya, 2017-. (PDF)

S1 Table Site specific sample size for vaccine effectiveness study of PCV-10 and start /end date of surveillance, Kenya, 2017–2024. (PDF)

S2 Table Sensitivity analysis of conjugate pneumococcal vaccine effectiveness calculated at sequential stages of study eligibility criteria, Kenya, 2017–2024. (PDF)

## References

[1] WHO. Health data overview for Kenya. 2025. Accessed 2025 May 20. https://data.who.int/countries/404

[2] Ministry of Health K. Kenya’s World Pneumonia Day Commitment. 2023. Accessed 2025 June 25. https://www.health.go.ke/kenyas-world-pneumonia-day-commitment

[3] Awori JO, Kamau A, Morpeth S, Kazungu S, Silaba M, Sande J. The etiology of pneumonia in HIV-uninfected children in Kilifi, Kenya: findings from the pneumonia etiology research for child health (PERCH) study. Pediatr Infect Dis J. 2021;40(9S):S29-39. doi: 10.1097/INF.0000000000002653 34448742 PMC8448399

[4] Pneumonia Etiology Research for Child Health (PERCH) Study Group. Causes of severe pneumonia requiring hospital admission in children without HIV infection from Africa and Asia: the PERCH multi-country case-control study. Lancet. 2019;394(10200):757–79. doi: 10.1016/S0140-6736(19)30721-4 31257127 PMC6727070

[5] Njuguna HN, Zaki SR, Roberts DJ, Rogena EA, Walong E, Fligner CL. Postmortem study of cause of death among children hospitalized with respiratory illness in Kenya. Pediatr Infect Dis J. 2021;40(8):715–22. doi: 10.1097/INF.0000000000003159 33967229 PMC8274582

[6] Feikin DR, Njenga MK, Bigogo G, Aura B, Aol G, Audi A, et al. Etiology and incidence of viral and bacterial acute respiratory illness among older children and adults in rural western Kenya, 2007-2010. PLoS One. 2012;7(8):e43656. doi: 10.1371/journal.pone.0043656 22937071 PMC3427162

[7] Sambala EZ, Wiyeh AB, Ngcobo N, Machingaidze S, Wiysonge CS. New vaccine introductions in Africa before and during the decade of vaccines - Are we making progress?. Vaccine. 2019;37(25):3290–5. doi: 10.1016/j.vaccine.2019.05.002 31076160

[8] Garcia Quesada M, Peterson ME, Bennett JC, Hayford K, Zeger SL, Yang Y, et al. Serotype distribution of remaining invasive pneumococcal disease after extensive use of ten-valent and 13-valent pneumococcal conjugate vaccines (the PSERENADE project): a global surveillance analysis. Lancet Infect Dis. 2025;25(4):445–56. doi: 10.1016/S1473-3099(24)00588-7 39706205 PMC11947070

[9] Alderson MR, Sethna V, Newhouse LC, Lamola S, Dhere R. Development strategy and lessons learned for a 10-valent pneumococcal conjugate vaccine (PNEUMOSIL®). Hum Vaccin Immunother. 2021;17(8):2670–7. doi: 10.1080/21645515.2021.1874219 33625961 PMC8475595

[10] Kobayashi M, Bigogo G, Kim L, Mogeni OD, Conklin LM, Odoyo A, et al. Impact of 10-valent pneumococcal conjugate vaccine introduction on pneumococcal carriage and antibiotic susceptibility patterns among children aged <5 years and adults with human immunodeficiency virus infection: Kenya, 2009-2013. Clin Infect Dis. 2020;70(5):814–26. doi: 10.1093/cid/ciz285 30959526 PMC6942635

[11] Hammitt LL, Akech DO, Morpeth SC, Karani A, Kihuha N, Nyongesa S, et al. Population effect of 10-valent pneumococcal conjugate vaccine on nasopharyngeal carriage of Streptococcus pneumoniae and non-typeable Haemophilus influenzae in Kilifi, Kenya: findings from cross-sectional carriage studies. Lancet Glob Health. 2014;2(7):e397-405. doi: 10.1016/S2214-109X(14)70224-4 25103393 PMC5628631

[12] Silaba M, Ooko M, Bottomley C, Sande J, Benamore R, Park K, et al. Effect of 10-valent pneumococcal conjugate vaccine on the incidence of radiologically-confirmed pneumonia and clinically-defined pneumonia in Kenyan children: an interrupted time-series analysis. Lancet Glob Health. 2019;7(3):e337–46. doi: 10.1016/S2214-109X(18)30491-1 30784634 PMC6379823

[13] Bigogo GM, Audi A, Auko J, Aol GO, Ochieng BJ, Odiembo H, et al. Indirect effects of 10-valent pneumococcal conjugate vaccine against adult pneumococcal pneumonia in rural western Kenya. Clin Infect Dis. 2019;69(12):2177–84. doi: 10.1093/cid/ciz139 30785189 PMC6861607

[14] Verani JR, Baqui AH, Broome CV, Cherian T, Cohen C, Farrar JL, et al. Case-control vaccine effectiveness studies: Preparation, design, and enrollment of cases and controls. Vaccine. 2017;35(25):3295–302. doi: 10.1016/j.vaccine.2017.04.037 28442231 PMC7007298

[15] Verani JR, Eno EN, Hunsperger EA, Munyua P, Osoro E, Marwanga D, et al. Acute febrile illness in Kenya: Clinical characteristics and pathogens detected among patients hospitalized with fever, 2017-2019. PLoS One. 2024;19(8):e0305700. doi: 10.1371/journal.pone.0305700 39088453 PMC11293630

[16] Ministry of Health K. Kenya National Immunization Policy Guidelines Version Signed. 2023. Accessed 2025 May 7. http://guidelines.health.go.ke:8000/media/Kenya_National_Immunization_Policy_Guidelines_Version_signed.pdf

[17] Greenland S, Pearl J, Robins JM. Causal diagrams for epidemiologic research. Epidemiology. 1999;10(1):37. 9888278

[18] Carey VJ. Gee: generalized estimation equation solver. 1999. Accessed 2025 May 30. https://CRAN.R-project.org/package=gee

[19] Knudson C. An introduction to model-fitting with the R package glmm. 2022.

[20] McCollum ED, Nambiar B, Deula R, Zadutsa B, Bondo A, King C, et al. Impact of the 13-valent pneumococcal conjugate vaccine on clinical and hypoxemic childhood pneumonia over three years in central Malawi: an observational study. PLoS One. 2017;12(1):e0168209. doi: 10.1371/journal.pone.0168209 28052071 PMC5215454

[21] Blyth CC, Britton KJ, Nguyen CD, Sapura J, Kave J, Nivio B, et al. Effectiveness of 13-valent pneumococcal conjugate vaccine against hypoxic pneumonia and hospitalisation in Eastern Highlands Province, Papua New Guinea: an observational cohort study. Lancet Reg Health West Pac. 2022;22:100432. doi: 10.1016/j.lanwpc.2022.100432 35308576 PMC8927990

[22] Tregnaghi MW, Sáez-Llorens X, López P, Abate H, Smith E, Pósleman A, et al. Efficacy of pneumococcal nontypable Haemophilus influenzae protein D conjugate vaccine (PHiD-CV) in young Latin American children: A double-blind randomized controlled trial. PLoS Med. 2014;11(6):e1001657. doi: 10.1371/journal.pmed.1001657 24892763 PMC4043495

[23] Weaver R, Nguyen CD, Chan J, Vilivong K, Lai JYR, Lim R, et al. The effectiveness of the 13-valent pneumococcal conjugate vaccine against hypoxic pneumonia in children in Lao People’s Democratic Republic: an observational hospital-based test-negative study. Lancet Reg Health West Pac. 2020;2:100014. doi: 10.1016/j.lanwpc.2020.100014 34327372 PMC8315332

[24] Hooli S, King C, McCollum ED, Colbourn T, Lufesi N, Mwansambo C, et al. In-hospital mortality risk stratification in children aged under 5 years with pneumonia with or without pulse oximetry: A secondary analysis of the Pneumonia REsearch Partnership to Assess WHO REcommendations (PREPARE) dataset. Int J Infect Dis. 2023;129:240–50. doi: 10.1016/j.ijid.2023.02.005 36805325 PMC10017350

[25] WHO. Immunization Data. WHO Immunization Data portal - African Region. Accessed 2025 May 20. https://immunizationdata.who.int/dashboard/regions/african-region

[26] Nsubuga P, White ME, Thacker SB, Anderson MA, Blount SB, Broome CV. Public health surveillance: a tool for targeting and monitoring interventions. In: Jamison DT, Breman JG, Measham AR, Alleyne G, Claeson M, Evans DB, eds. Disease control priorities in developing countries. 2nd ed. Washington (DC): The International Bank for Reconstruction and Development / The World Bank. 2006. 21250309

[27] Field E, Strathearn M, Boyd-Skinner C, Dyda A. Usefulness of linked data for infectious disease events: a systematic review. Epidemiol Infect. 2023;151:e46. doi: 10.1017/S0950268823000316 PMC10052405 36843485

[28] Kenya Ministry of Health. Technical guidelines for integrated disease surveillance and response. 2022. https://nphi.go.ke/sites/default/files/2024-02/IDSR%20Technical%20Guidelines%20for%20Kenya%2011.05.2022_9am.pdf

[29] Centers for Disease Control and Prevention. Chikungunya in the Region of the Indian Ocean - Level 2 - Practice Enhanced Precautions - Travel Health Notices. 2025. Accessed 2025 August 14. https://wwwnc.cdc.gov/travel/notices/level2/chikungunya-indian-ocean-islands

[30] ICAP Global Health. ICAP-Trained Surveillance Team Identifies Human Rift Valley Fever Case in Marsabit, Kenya. 2024. Accessed 2025 August 14. https://icap.columbia.edu/news-events/icap-trained-surveillance-team-identifies-human-rift-valley-fever-case-in-marsabit-kenya/

[31] Itoh M, Lucchi N, Schultz J, Agogo G, Munyua P, Chege D. Using sentinel surveillance system data to characterize severe malaria illness and quality of malaria case management among hospitalized patients in Kenya, 2017-2024. Malar J. 2026;25(1):90. doi: 10.1186/s12936-025-05738-3 41535861 PMC12888659

[32] Johnson CN, Wilde S, Tuomanen E, Rosch JW. Convergent impact of vaccination and antibiotic pressures on pneumococcal populations. Cell Chem Biol. 2024;31(2):195–206. doi: 10.1016/j.chembiol.2023.11.003 38052216 PMC10938186

[33] Prendergast AJ. Malnutrition and vaccination in developing countries. Philos Trans R Soc Lond B Biol Sci. 2015;370(1671):20140141. doi: 10.1098/rstb.2014.0141 25964453 PMC4527386

[34] Cohen C, von Mollendorf C, de Gouveia L, Naidoo N, Meiring S, Quan V. Effectiveness of 7-valent pneumococcal conjugate vaccine against invasive pneumococcal disease in HIV-infected and -uninfected children in south africa: a matched case-control study. Clin Infect Dis. 2014;59(6):808–18. doi: 10.1093/cid/ciu431 24917657 PMC4144265

[35] Nayakwadi Singer M, Heath C, Muinde J, Gildengorin V, Mutuku FM, Vu D, et al. Pneumococcal vaccine response after exposure to parasites in utero, in infancy, or mid-childhood. Pediatrics. 2017;139(4):e20162781. doi: 10.1542/peds.2016-2781 28302673 PMC5369673

