# Supplementary material for: Pneumococcal conjugate vaccine effectiveness against hypoxemia in children with suspected pneumonia in Kenya; analysis from a real-world sentinel surveillance platform
Source: PLoS One. 2026 Jun 26;21(6):e0351500. doi: 10.1371/journal.pone.0351500 (PMC13308777; doi:10.1371/journal.pone.0351500)

S2 Fig. Study flow chart for vaccine effectiveness study of PCV-10 using sentinel surveillance data in Kenya, 2017-2024

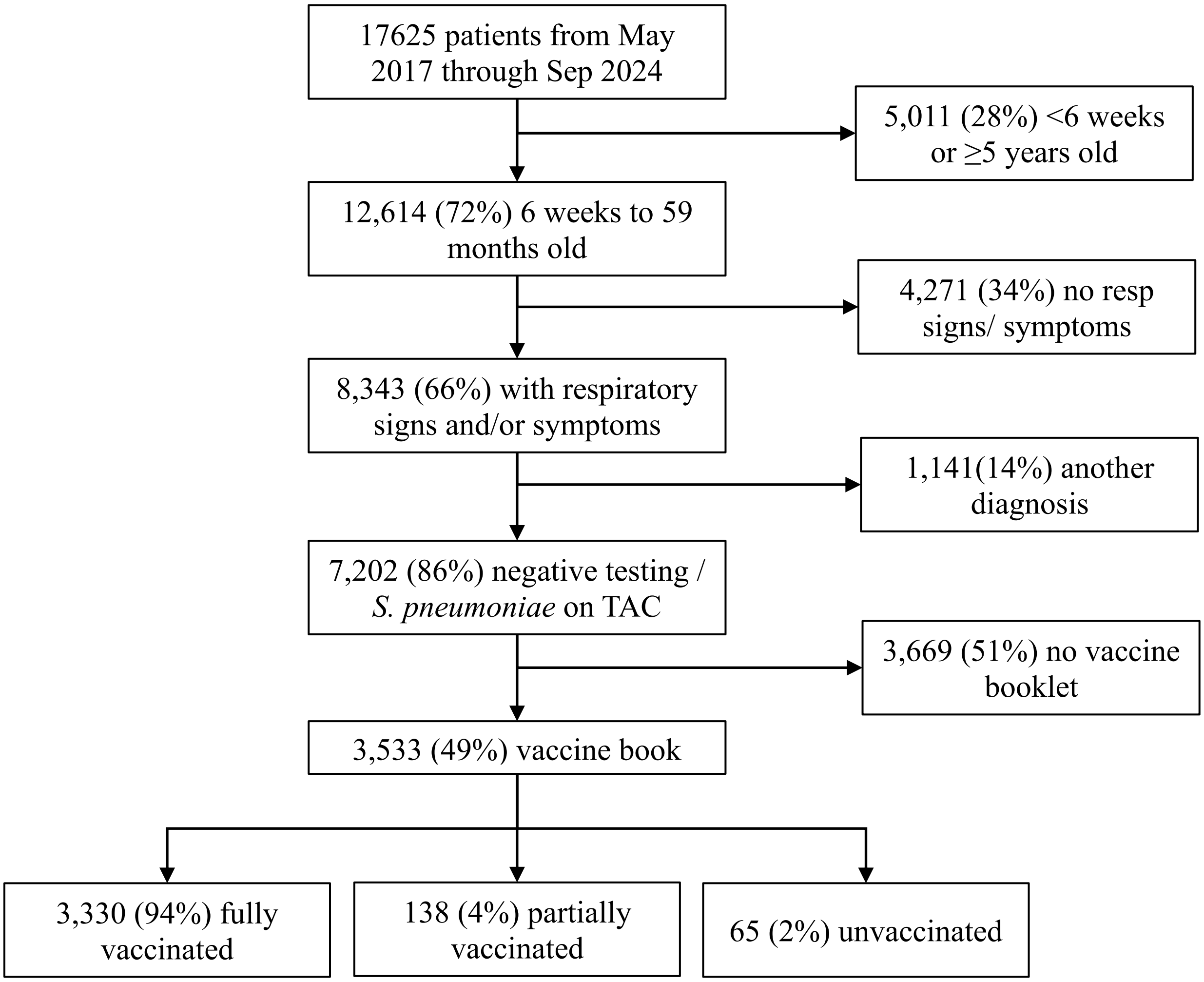

Supplement: S2 Fig — (PDF) [file pone.0351500.s002.pdf]
