# Supplementary material for: Pneumococcal conjugate vaccine effectiveness against hypoxemia in children with suspected pneumonia in Kenya; analysis from a real-world sentinel surveillance platform
Source: PLoS One. 2026 Jun 26;21(6):e0351500. doi: 10.1371/journal.pone.0351500 (PMC13308777; doi:10.1371/journal.pone.0351500)
Supplement: S1 Table — (PDF) [file pone.0351500.s003.pdf]

S1 Table. Years of data collection by site for vaccine effectiveness study of pneumococcal conjugate vaccine, Kenya, 2017-2024

<table>
<tr><th></th><th>Site name</th><th>County</th><th>2017</th><th>2018</th><th>2019</th><th>2020</th><th>2021</th><th>2022</th><th>2023</th><th>2024</th></tr>
<tr><td>1</td><td>Busia County Referral Hospital</td><td>Busia</td><td></td><td></td><td></td><td></td><td style="background:#bfbfbf"></td><td style="background:#bfbfbf"></td><td style="background:#bfbfbf"></td><td style="background:#bfbfbf"></td></tr>
<tr><td>2</td><td>Coast General Teaching and Referral Hospital</td><td>Mombasa</td><td style="background:#bfbfbf"></td><td style="background:#bfbfbf"></td><td style="background:#bfbfbf"></td><td style="background:#bfbfbf"></td><td style="background:#bfbfbf"></td><td style="background:#bfbfbf"></td><td style="background:#bfbfbf"></td><td style="background:#bfbfbf"></td></tr>
<tr><td>3</td><td>Jaramogi Oginga Odinga Teaching and Referral Hospital</td><td>Kisumu</td><td></td><td></td><td></td><td></td><td></td><td style="background:#bfbfbf"></td><td style="background:#bfbfbf"></td><td style="background:#bfbfbf"></td></tr>
<tr><td>4</td><td>Kakamega County Referral Hospital</td><td>Kakamega</td><td></td><td style="background:#bfbfbf"></td><td style="background:#bfbfbf"></td><td style="background:#bfbfbf"></td><td style="background:#bfbfbf"></td><td></td><td></td><td></td></tr>
<tr><td>5</td><td>Kakuma Refugee Camp Hospital</td><td>Turkana</td><td style="background:#bfbfbf"></td><td style="background:#bfbfbf"></td><td style="background:#bfbfbf"></td><td style="background:#bfbfbf"></td><td style="background:#bfbfbf"></td><td></td><td></td><td></td></tr>
<tr><td>6</td><td>Kapenguria County Referral Hospital</td><td>West Pokot</td><td></td><td></td><td></td><td></td><td style="background:#bfbfbf"></td><td style="background:#bfbfbf"></td><td style="background:#bfbfbf"></td><td style="background:#bfbfbf"></td></tr>
<tr><td>7</td><td>Kenyatta National Hospital</td><td>Nairobi</td><td style="background:#bfbfbf"></td><td style="background:#bfbfbf"></td><td style="background:#bfbfbf"></td><td style="background:#bfbfbf"></td><td style="background:#bfbfbf"></td><td style="background:#bfbfbf"></td><td style="background:#bfbfbf"></td><td></td></tr>
<tr><td>8</td><td>Loitokitok Subcounty Hospital</td><td>Kajiado</td><td></td><td></td><td></td><td></td><td style="background:#bfbfbf"></td><td style="background:#bfbfbf"></td><td style="background:#bfbfbf"></td><td style="background:#bfbfbf"></td></tr>
<tr><td>9</td><td>Makueni County Referral Hospital</td><td>Makueni</td><td></td><td></td><td></td><td></td><td style="background:#bfbfbf"></td><td style="background:#bfbfbf"></td><td style="background:#bfbfbf"></td><td style="background:#bfbfbf"></td></tr>
<tr><td>10</td><td>Mama Lucy Hospital</td><td>Nairobi</td><td></td><td></td><td></td><td></td><td style="background:#bfbfbf"></td><td style="background:#bfbfbf"></td><td style="background:#bfbfbf"></td><td style="background:#bfbfbf"></td></tr>
<tr><td>11</td><td>Marsabit County Referral Hospital</td><td>Marsabit</td><td></td><td></td><td></td><td style="background:#bfbfbf"></td><td style="background:#bfbfbf"></td><td style="background:#bfbfbf"></td><td style="background:#bfbfbf"></td><td style="background:#bfbfbf"></td></tr>
<tr><td>12</td><td>Migori County Referral Hospital</td><td>Migori</td><td></td><td></td><td></td><td></td><td></td><td></td><td></td><td style="background:#bfbfbf"></td></tr>
<tr><td>13</td><td>Nakuru County Referral Hospital</td><td>Nakuru</td><td></td><td></td><td></td><td></td><td style="background:#bfbfbf"></td><td style="background:#bfbfbf"></td><td style="background:#bfbfbf"></td><td style="background:#bfbfbf"></td></tr>
</table>
