# Supplementary material for: Pneumococcal conjugate vaccine effectiveness against hypoxemia in children with suspected pneumonia in Kenya; analysis from a real-world sentinel surveillance platform
Source: PLoS One. 2026 Jun 26;21(6):e0351500. doi: 10.1371/journal.pone.0351500 (PMC13308777; doi:10.1371/journal.pone.0351500)
Supplement: S2 Table — (PDF) [file pone.0351500.s004.pdf]

S2 Table. Sensitivity analysis of conjugate pneumococcal vaccine effectiveness calculated at sequential stages of study eligibility criteria, Kenya, 2017-2024

Metric	Age 6w–59m	Respiratory features present	Negative TAC, Malaria, SC2*	Child health booklet
N	12,614	8,343	7,202	3,533
% fully vaccinated	95.8	95.0	94.9	94.3
VE% (95% CI) Univariate: Full PCV Vaccination	39.6 (17.8–55.2)	37.9 (12.4–55.7)	34.5 (5.7–54.3)	46.6 (12.6–67.5)
(Δlog OR)^2 vs final, univariate	0.0151	0.0229	0.0419	-
|ΔVE| vs final (pp), univariate	7.0	8.7	12.1	-

* A negative test result on multiplex TAC PCR, malaria microscopy or rapid diagnostic test, or SARS-CoV-2 PCR.

PCV: Pneumococcal conjugate vaccine; PCR: polymerase chain reaction; SC2: SARS-Cov-2; TAC: TaqMan Array Card; VE: Vaccine effectiveness
