# Supplementary material for: Pneumococcal conjugate vaccine effectiveness against hypoxemia in children with suspected pneumonia in Kenya; analysis from a real-world sentinel surveillance platform
Source: PLoS One. 2026 Jun 26;21(6):e0351500. doi: 10.1371/journal.pone.0351500 (PMC13308777; doi:10.1371/journal.pone.0351500)
Supplement: Attachment — Submitted filename: Comments for HINES.docx [file pone.0351500.s005.docx]

General:

A very convincing, succinct paper. Two or three things might warrant additional comment:

In the discussion—can anything be said about HIV positive children, or are there too few?

It’s interesting that this is one of the highest VE values from RWE reported (per your discussion). Any comment?

Can you comment on the nutritional/MUAC data? Is this possibly related to having a chronic disease such as HIV, or is this an overwhelming effect of malnutrition wiping out vaccine benefits?

Line 96—for the past 24 hr.

On Monday, were the records also reviewed and included for the weekend admissions?

Line 102—please define AFI at first use

Line 109--(either by verbal report or transcribed from the Ministry of Health child health booklet),

Just to be clear, the inclusion criteria were described as requiring the MOH Child Health booklet, but it was not necessarily used to collect the data. Is this correct?

Line 113--(Masimo Rad-5 and Lifebox®)

Were data generated to support the comparability of measurements from these 2 devices?

Line 184—Patients >8 years

Were patients greater than 8 years excluded or patients greater than 59 months? please explain

Line	203--Blood specimens were positive for S. pneumoniae

For clarity, mention the test. Was this the TAC PCR test?

Line 206--The percentage of hypoxemic patients who were fully 206 vaccinated was 93.2% compared to 94.9% in those without hypoxemia.

This difference is small; does that call into question the conclusion of the paper?

Lines 242-252

Would rearranging this paragraph to start with all discussion of pneumonia followed by hypoxemic pneumonia make it easier to follow?

Line 308--Nevertheless, we contend that the value of obtaining real-world evidence outweighs this limitation

Agreed, and the fact that it is contemporary evidence is very important!
