# Supplementary material for: Pneumococcal conjugate vaccine effectiveness against hypoxemia in children with suspected pneumonia in Kenya; analysis from a real-world sentinel surveillance platform
Source: PLoS One. 2026 Jun 26;21(6):e0351500. doi: 10.1371/journal.pone.0351500 (PMC13308777; doi:10.1371/journal.pone.0351500)
Supplement: Attachment — Submitted filename: Response to reviewers - PONE-D-25-49348.docx [file pone.0351500.s006.docx]

18 April 2026

Dear PLOS One editor,

Thank you for the opportunity to revise and resubmit our revised manuscript, “Pneumococcal conjugate vaccine effectiveness against hypoxemia in children with suspected pneumonia in Kenya; analysis from a real-world sentinel surveillance platform.

PLOS One” (PONE-D-25-49348). Please find enclosed an updated manuscript with changes made in response to the reviewers’ detailed comments and edits, as well as our point-by-point replies to the comments below. We hope that these changes address the journal editor’s decision and that the article meets the high standards of PLOS One journal. We look forward to hearing back from you.

Sincerely,

Jonas Hines

**Journal Requirements:**

When submitting your revision, we need you to address these additional requirements.

1. Please ensure that your manuscript meets PLOS ONE's style requirements, including those for file naming. The PLOS ONE style templates can be found at

[https://journals.plos.org/plosone/s/file?id=wjVg/PLOSOne_formatting_sample_main_body.pdf](https://track.editorialmanager.com/CL0/https:%2F%2Fjournals.plos.org%2Fplosone%2Fs%2Ffile%3Fid=wjVg%2FPLOSOne_formatting_sample_main_body.pdf/1/010f019d41e852ff-f80730a1-0497-4756-bcb6-8b3651bfb75b-000000/Kgl1poXpsyfalFdOiPnFBBRG6bsjvRlwytapyRcsrWM=256) and

[https://journals.plos.org/plosone/s/file?id=ba62/PLOSOne_formatting_sample_title_authors_affiliations.pdf](https://track.editorialmanager.com/CL0/https:%2F%2Fjournals.plos.org%2Fplosone%2Fs%2Ffile%3Fid=ba62%2FPLOSOne_formatting_sample_title_authors_affiliations.pdf/1/010f019d41e852ff-f80730a1-0497-4756-bcb6-8b3651bfb75b-000000/Nh1thy5VfQAf-LhGPaOvTex2ItA6EQaRO8xI1MKZ0q4=256)

*Authors reply*: We have updated files and file names to comply with PLOS ONE styles.

2. We note that the grant information you provided in the ‘Funding Information’ and ‘Financial Disclosure’ sections do not match.

When you resubmit, please ensure that you provide the correct grant numbers for the awards you received for your study in the ‘Funding Information’ section.

*Authors reply*: We have confirmed the grant numbers are accurate in the financial information section.

3. Please note that funding information should not appear in any section or other areas of your manuscript. We will only publish funding information present in the Funding Statement section of the online submission form. Please remove any funding-related text from the manuscript.

*Authors reply*: We have removed this information from the manuscript file.

4. For studies involving third-party data, we encourage authors to share any data specific to their analyses that they can legally distribute. PLOS recognizes, however, that authors may be using third-party data they do not have the rights to share. When third-party data cannot be publicly shared, authors must provide all information necessary for interested researchers to apply to gain access to the data. (<https://journals.plos.org/plosone/s/data-availability#loc-acceptable-data-access-restrictions>)

For any third-party data that the authors cannot legally distribute, they should include the following information in their Data Availability Statement upon submission:

1) A description of the data set and the third-party source

2) If applicable, verification of permission to use the data set

3) Confirmation of whether the authors received any special privileges in accessing the data that other researchers would not have

4) All necessary contact information others would need to apply to gain access to the data

*Authors reply*: We have updated the data availability statement to address this comment and provide information on how other researchers can request data access. The statement now reads, “The data are bound by Government of Kenya provisions, including the Data Protection Act of 2019. This data was accessed via an approved Data Sharing Agreement with ICAP, the data steward. Data access requests require the permission of Kenya Ministry of Health and can be directed to [dc2929@cumc.columbia.edu](mailto:dc2929@cumc.columbia.edu).”

5. Please include captions for your Supporting Information files at the end of your manuscript, and update any in-text citations to match accordingly. Please see our Supporting Information guidelines for more information: [http://journals.plos.org/plosone/s/supporting-information](http://track.editorialmanager.com/CL0/http:%2F%2Fjournals.plos.org%2Fplosone%2Fs%2Fsupporting-information/1/010f019d41e852ff-f80730a1-0497-4756-bcb6-8b3651bfb75b-000000/z_-14EGZSDyzBuVFf_GRt9k9cPBufjpFnoCn1V_qTiI=256).

*Authors reply*: We have added the supplemental material to the supporting information section in the manuscript and updated the file names. Please note, we changed one supplemental figure to a main text figure.

6. We note that Supplementary Figure 1 in your submission contain map images which may be copyrighted. All PLOS content is published under the Creative Commons Attribution License (CC BY 4.0), which means that the manuscript, images, and Supporting Information files will be freely available online, and any third party is permitted to access, download, copy, distribute, and use these materials in any way, even commercially, with proper attribution. For these reasons, we cannot publish previously copyrighted maps or satellite images created using proprietary data, such as Google software (Google Maps, Street View, and Earth). For more information, see our copyright guidelines: <http://journals.plos.org/plosone/s/licenses-and-copyright>.

We require you to either (1) present written permission from the copyright holder to publish these figures specifically under the CC BY 4.0 license, or (2) remove the figures from your submission:

1. You may seek permission from the original copyright holder of Supplementary Figure 1 to publish the content specifically under the CC BY 4.0 license.

We recommend that you contact the original copyright holder with the Content Permission Form (<http://journals.plos.org/plosone/s/file?id=7c09/content-permission-form.pdf>) and the following text:

“I request permission for the open-access journal PLOS ONE to publish XXX under the Creative Commons Attribution License (CCAL) CC BY 4.0 (<http://creativecommons.org/licenses/by/4.0/>). Please be aware that this license allows unrestricted use and distribution, even commercially, by third parties. Please reply and provide explicit written permission to publish XXX under a CC BY license and complete the attached form.”

Please upload the completed Content Permission Form or other proof of granted permissions as an "Other" file with your submission.

In the figure caption of the copyrighted figure, please include the following text: “Reprinted from [ref] under a CC BY license, with permission from [name of publisher], original copyright [original copyright year].”

2. If you are unable to obtain permission from the original copyright holder to publish these figures under the CC BY 4.0 license or if the copyright holder’s requirements are incompatible with the CC BY 4.0 license, please either i) remove the figure or ii) supply a replacement figure that complies with the CC BY 4.0 license. Please check copyright information on all replacement figures and update the figure caption with source information. If applicable, please specify in the figure caption text when a figure is similar but not identical to the original image and is therefore for illustrative purposes only.

The following resources for replacing copyrighted map figures may be helpful:

USGS National Map Viewer (public domain): <http://viewer.nationalmap.gov/viewer/>

The Gateway to Astronaut Photography of Earth (public domain): <http://eol.jsc.nasa.gov/sseop/clickmap/>

Maps at the CIA (public domain): <https://www.cia.gov/library/publications/the-world-factbook/index.html> and <https://www.cia.gov/library/publications/cia-maps-publications/index.html>

NASA Earth Observatory (public domain): <http://earthobservatory.nasa.gov/>

Landsat: <http://landsat.visibleearth.nasa.gov/>

USGS EROS (Earth Resources Observatory and Science (EROS) Center) (public domain): [http://eros.usgs.gov/#](http://eros.usgs.gov/)

Natural Earth (public domain): [http://www.naturalearthdata.com/](http://track.editorialmanager.com/CL0/http:%2F%2Fwww.naturalearthdata.com%2F/1/010f019d41e852ff-f80730a1-0497-4756-bcb6-8b3651bfb75b-000000/YfM9F5G-eGg5OBwqp5gjFZoutX5DpZJrZlKxZDbF6oE=256)

*Authors reply*: We recreated the figure in R to use shapefiles from sources that have a CC license. The sources of our shapefiles are:

1. Map of Africa: <https://www.naturalearthdata.com/downloads/10m-cultural-vectors/10m-admin-0-details/>
2. Map of Kenya (counties): <https://data.humdata.org/dataset/cod-ab-ken>
3. Lakes: <https://energydata.info/dataset/africa-water-bodies>).

7. If the reviewer comments include a recommendation to cite specific previously published works, please review and evaluate these publications to determine whether they are relevant and should be cited. There is no requirement to cite these works unless the editor has indicated otherwise.

*Authors reply*: There were no such reviewer comments.

8. Please review your reference list to ensure that it is complete and correct. If you have cited papers that have been retracted, please include the rationale for doing so in the manuscript text, or remove these references and replace them with relevant current references. Any changes to the reference list should be mentioned in the rebuttal letter that accompanies your revised manuscript. If you need to cite a retracted article, indicate the article’s retracted status in the References list and also include a citation and full reference for the retraction notice.

*Authors reply*: We have reviewed the citations to confirm none have been retracted. One citation (#31) that was a pre-print was replaced with the now published article.

**Review Comments to the Author**
Reviewer #1: Thank you for the opportunity to review “Pneumococcal conjugate vaccine effectiveness against hypoxemia in children with suspected pneumonia in Kenya; analysis from a real-world sentinel surveillance platform”. This is a nice, well written paper that will add to the literature on this topic. I have a few minor comments for the authors to consider:

What is AFI? Please spell out all your acronyms where they first appear (e.g. AFI appears on line 102)

*Authors reply*: We mistakenly omitted the acronym definition on line 94, which has now been updated. Thank you for catching this.

What was enrolment rate into surveillance study? I see that 17,625 were enrolled, but how many refused enrollment or were otherwise not enrolled? E.g., You note in your limitations section that your enrolling scheme may have missed deaths; can you quantify this and other potential ‘exclusions’? If not, please make this explicit in your results and limitations.

*Authors reply*:

We have added text about the eligibility and enrollment to the beginning of the results, which now reads, “From May 27, 2017 to September 5, 2024, out of 151,546 patients screened , 23,774 (16%) were eligible, of whom 17,625 (74%) were enrolled (percent enrolled range: 58% [2017] to 82% [2021]).”

Additionally, to clarify the second point by the reviewer, we added the text to lines 109-110, “Patients who died before they could be approached by surveillance officers were not eligible for enrolment.” to the description of the surveillance system protocol to ensure this detail is well captured. Additionally, we reinforce this limitation at lines 328-330 ,stating, “Recruiting patients the morning after admission likely excluded those who died soon after presentation to the health facility, biasing toward less severe cases in the surveillance system.”

Methods line 109, results line 196. In your methods you report verbal report for data collection, but in the results, you only include verifiable results from a vaccination record. Please clarify, perhaps in your Data Analysis section.

*Authors reply*: The surveillance officers document vaccination history preferably from the child health booklet but by participant self-report if the booklet is unavailable. To reduce the risk of bias in our analysis, we restricted to patients who had PCV-10 vaccine history documented in their child health book, as pointed out in this reviewer’s comment. The paragraph from line 154-163 in the data analysis section explains the exclusion criteria for the analysis, including that we “excluded patients with missing vaccination information or vaccination history that was by verbal report alone.”

Results, line 205+: This statement is a little unintuitive, and I’m not sure you wish to show the data like this? Consider instead something like: “Hypoxemia was less common among fully vaccinated patients compared to unvaccinated patients ()”. The percentages differ only a little, without seeing the absolute numbers or even a p value, it is difficult to understand if these are meaningful differences. To my eye, 93.2% compared to 94.9% is nearly the same.

*Authors reply*: We agree with this comment. The difference in vaccine coverage based on hypoxemia status is small, reflecting the overall high PCV-10 vaccine coverage in the study population. However, 34/65 unvaccinated children were hypoxemic compared to 1,230/3,330 fully vaccinated children (52% vs. 37%). The distinction in these two estimates is in whether column percent (as displayed in Table 1) or row percent are presented. We have removed this sentence from the results narrative since the row percent is presented in the following paragraph; however, the column percent remains in Table 1.

Table 1: how is “absconded” different from “missing”?

*Authors reply*: Missing refers to missing data on the patient’s outcome whereas absconded is an actual patient outcome in Kenya (equivalent to leaving against medical advice).

Comments from Editor

General: A very convincing, succinct paper. Two or three things might warrant additional comment:

In the discussion—can anything be said about HIV positive children, or are there too few?

*Authors reply*: Unfortunately, there were too few patients who reported being HIV positive to do more in-depth analysis. HIV status was self-reported and HIV prevalence among children in Kenya is low (0.4% in 0-4 year-olds in the KENPHIA 2018 [<https://journals.plos.org/plosone/article?id=10.1371/journal.pone.0277613>]), and HIV history in IFBS is self-reported.

It’s interesting that this is one of the highest VE values from RWE reported (per your discussion). Any comment?

*Authors reply*: We agree that the VE estimate for hypoxemic pneumonia in this study was greater but cautiously compare point estimates between studies with different methodology. these studies point in the same direction of PCV-10 effectiveness is notable itself. We have added the sentence, “Our findings add to the evidence of effectiveness of PCV-10 against pneumonia,” to better describe the topic of this paragraph.

Can you comment on the nutritional/MUAC data? Is this possibly related to having a chronic disease such as HIV, or is this an overwhelming effect of malnutrition wiping out vaccine benefits?

*Authors reply*: Malnutrition decreases vaccine effectiveness. Although malnutrition is prevalent among CLHIV, our data does not allow us to investigate the relationship between malnutrition and HIV. We have added an additional citation that supports the relationship between malnutrition and reduced immunogenicity of PCV-10.

Line 96—for the past 24 hr.

On Monday, were the records also reviewed and included for the weekend admissions?

*Authors reply*: Because the surveillance platform does not operate on weekends, on Monday mornings surveillance officers review admission registers for weekend admissions and approach eligible patients if they meet inclusion criteria and have received antimicrobial therapy for < 24 hours. This approach balances comprehensive case capture with the reduced diagnostic yield after antimicrobial treatment begins. We have updated the description of the surveillance platform with the following sentence: “Because surveillance was not conducted on weekends, surveillance officers also reviewed weekend admissions on Monday mornings and approached eligible patients for enrolment if they had received antimicrobial therapy for less than 24 hours.”

Line 102—please define AFI at first use

*Authors reply*: Please see the above reviewer 1 comment and our reply that address this comment.

Line 109--(either by verbal report or transcribed from the Ministry of Health child health booklet),

Just to be clear, the inclusion criteria were described as requiring the MOH Child Health booklet, but it was not necessarily used to collect the data. Is this correct?

*Authors reply*: This is correct. We restricted to patients who had vaccine history documented from their child health book. S2 Table contains a sensitivity analysis that includes patients regardless of the source of their vaccination history.

Line 113--(Masimo Rad-5 and Lifebox®)

Were data generated to support the comparability of measurements from these 2 devices?

*Authors reply*: King et al. established comparability between Masimo and Lifebox devices in Malawi (<https://onlinelibrary.wiley.com/doi/full/10.1002/ppul.24295>). This project did not directly compare devices.

Line 184—Patients >8 years

Were patients greater than 8 years excluded or patients greater than 59 months? please explain

*Authors reply*: This refers to the general IFBS consenting process since all ages are eligible for enrolment in the surveillance system. We’ve updated this section to remove the ±8yo cut-off to avoid confusion. The sentence now reads: “During eligibility screening for enrolment in the IFBS system, the surveillance officer obtained verbal and written informed consent in English, Swahili, or a preferred local language from a parent or guardian of patients, including for data collection and biological specimens.”

Line	203--Blood specimens were positive for S. pneumoniae

For clarity, mention the test. Was this the TAC PCR test?

*Authors reply*: We have added “on TAC PCR test” to clarify this detail.

Line 206--The percentage of hypoxemic patients who were fully 206 vaccinated was 93.2% compared to 94.9% in those without hypoxemia.

This difference is small; does that call into question the conclusion of the paper?

*Authors reply*: Please see the above reviewer 1 comment and our reply that address this comment. We appreciate that how we presented this result was potentially confusing.

Lines 242-252

Would rearranging this paragraph to start with all discussion of pneumonia followed by hypoxemic pneumonia make it easier to follow?

*Authors reply*: We have rearranged the studies presented in this paragraph to incorporate this comment.

Line 308--Nevertheless, we contend that the value of obtaining real-world evidence outweighs this limitation

Agreed, and the fact that it is contemporary evidence is very important!

*Authors reply*: Thank you for this comment.
